# A Corpus Linguistics Approach to the Representation of Western Religious Beliefs in Ten Series of Chinese University English Language Teaching Textbooks

**DOI:** 10.3389/fpsyg.2021.789660

**Published:** 2022-01-20

**Authors:** Yanhong Liu, Lawrence Jun Zhang, Li Yang

**Affiliations:** ^1^School of Foreign Studies, Yanshan University, Qinghuangdao, China; ^2^Faculty of Education and Social Work, University of Auckland, Auckland, New Zealand; ^3^School of Foreign Languages, Jiangsu University of Science and Technology, Zhenjiang, China

**Keywords:** religion, ELT textbooks, corpus linguistics, critical discourse analysis, cultural representation

## Abstract

The early Sino-Western contact was through the way in which religion and language interact to produce language contact. However, research on this contact is relatively limited to date, particularly in the realm of English language materials. In fact, there is a paucity of research on Western religions in English Language Teaching (ELT) textbooks. By applying corpus linguistics as a tool and the Critical Discourse Analysis as the theoretical framework, this manuscript critically investigates the significant semantic domains in ten English language textbook series that are officially approved and are widely used in Chinese universities. The findings suggest that various Western religious beliefs, which are the highly unusual topics in previous Chinese ELT textbooks, are represented in the textbook corpus. The results also show that when presenting the views and attitudes toward Western religious beliefs, these textbooks have adopted an eclectic approach to the material selection. Surprisingly, positive semantic prosody surrounding the concept of religion is evident and no consistent negative authorial stance toward religion is captured. Atheism has been assumed to be in the center of Chinese intellectual traditions and the essence of the Constitution of the Chinese Communist Party. Interestingly, the findings from this study provide a new understanding of Chinese foreign language textbooks in the new era, and its addition to the literature on the study of ELT textbooks, as well as its development worldwide.

## Introduction

Language and religion share a very long and close history (Sawyer, 2001), though their contact is rarely explored before in China (Spolsky, 2003). In the early 19th century, missionaries reached China and became the most immediate channel of Western knowledge, as well as the English language. Robert Morrison (1782–1834), a pioneer Protestant missionary, brought out a Chinese version of the Bible, which was later used by the leader of the Taiping Rebellion, and has influenced the early Taiping religious documents. Foremost among the pioneers, Young J. Allen created a Chinese periodical, the *Wan-kuo kung-pao* (“*The Globe Magazine*”) (1875–1907), and committed to “the extension of knowledge relating to Geography, History, Civilization, Politics, Religion, Science, Art, Industry, and General Progress of Western countries” (Teng and Fairbank, 1954, p. 134). The early missionaries’ activities had influence on Chinese people’s social practices. However, the acceptance of Western thoughts, specifically Western religions, was very low. This denial of, and rejection to, Western religion can be traced back to as early as the sixteenth century. The early Sino-Western contact was the conflict between the conviction of Confucianism and the ideas of Western religions.

Confucianism is the philosophical factor underlying the Chinese’ negative response to the Western civilization and thoughts. Chinese intellectuals’ conviction has its roots in Confucianism, and its orthodoxy, being the heart of Chinese civilization, has been established and is constantly preserved by Chinese people of all levels, particularly the gentry and the scholars. The first extensive cultural contact between China and Europe happened at the end of the sixteenth century through Jesuit missionaries in the wake of the Portuguese invasion. The Jesuits found it easier to influence China’s science than its religion. Being the upholders of Chinese traditional culture, most of the native scholars, entrenched in their ethnocentric cultural tradition, were not seriously affected by the new elements of Western thought (Teng and Fairbank, 1954). In addition, missionaries’ dual interests in the Chinese people were not only in “saving their souls” through Christianity, but also in improving their lives. Their efforts to plant ideas of reform in the minds of people they could reach were the source of resentment from the Chinese gentry-scholar-official class.

Although some Western missionaries’ ideas, including elements of mathematics, geography, astronomy, and calendar, were valued by the Chinese people, the gentry-officials had experienced the difficulty of separating Western religions from other aspects of Western civilization. They might have willingly accepted the latter even without the former had they seen a distinction (Teng and Fairbank, 1954). Thus, the missionaries at the Chinese court in the 1770s were limited to serving as technical personnel – painters, musicians, and architects – rather than as persons of intellectual importance. The freedom to propagate the faith was permitted, but priests were forbidden to interfere in Chinese internal politics or to attack Confucianism.

Emergent culture represents nascent ways of behavior that are still in the process of being shared and established (Williams, 1976). At the turn of the century, China’s socio-cultural transformations have witnessed some emerging ways of exploring Western philosophy and other ideas, as evidenced in the College English Curriculum Requirements (2007) (*Requirements* hereafter) issued by the Ministry of Education of China (MoE). For the purpose of improving national development and enriching global society, the Higher Education Department of Ministry of Education (2007) stipulated that “the objective of College English is to develop students’ ability to use English in a well-rounded way… and improve their general cultural awareness so as to meet the needs of China’s social development and international exchanges” (Higher Education Department of Ministry of Education, 2007, p. 23).

In 2010, the State Council of China launched the *State Planning Outline for Medium and Long-term Education Reform and Development* (*2010–2020*) (hereafter *Outline*), which addressed the direction and the strategies of educational reform and development. This *Outline* also made it clear that English education is of significance for internationalized education, particularly in the context of globalization.

In the era of globalization, under the influence of China’s Open Door reform policy and the new “Belt and Road Initiative,” China is actively connecting with the world. Given such a background, how has the phenomenon of negativity toward Western religions changed in the way that the English textbooks were written or compiled?By drawing on the synergy of the theoretical framework of Critical Discourse Analysis (CDA) and of corpus linguistics as a tool, this manuscript aims to examine whether Western religions are represented in Chinese university ELT textbooks, and if so, how are the religious beliefs being presented. As Risager (2021) posited, language textbooks may be developed in one country, while they can be used in another; the textbook users, teachers, and students may be located in different countries. Thus, the findings may offer relevant implications for English as a Foreign Language (EFL) teaching and learning, along with textbook development worldwide.

## Literature Review

### Language Textbook Analysis in China

The critical role of textbooks in English programs has been identified by an increasing number of academics (e.g., McDonough and Shaw, 1993; Cunningsworth, 1995; Tomlinson, 2001; Richards, 2006; Newton and Newton, 2009; Guilloteaux, 2013; Tsagari and Sifakis, 2014). Thus, studies into English language textbooks in other parts of the world have been published since the 1960s. In China, however, the investigation of language textbooks is still in its infancy, although China has the largest number of English learners, and the EFL/ESL learning and teaching heavily rely on textbooks (Liu, 2017). Indeed, it was not until the year 2000 that studies of English language textbooks began to attract some attention in China (Huang and Yu, 2009). White (1998) holds the view that textbooks serve language curriculums on three levels:

(1) Linguistic components: grammar, vocabulary, and language skills.
(2) Pedagogy: explicit and/or implicit beliefs and practices for teaching and learning.
(3) Content: situational contexts and topics, including political and moral messages.

Apart from the early studies discussing the textbook compiling criteria and guidelines (e.g., Qian, 1995; Zhao and Zheng, 2006), empirical studies into EFL/ESL textbooks focused exclusively on the first level – investigating linguistic items, focusing on the breadth and depth of vocabulary, collocation and pronunciation of middle school English textbooks (see He, 2007, 2009; Liang and He, 2009; Zheng and He, 2009), and the university English textbooks in China (see Liu and Liu, 2011a,b; Liu and Zhang, 2015; Zhang and Liu, 2015).

Regarding the “content,” several attempts have been made to investigate the cultural representations (e.g., Zhang and Zhu, 2002; Yuen, 2011; Liu et al., 2015, 2021) and ideological underpinnings (Price, 1971; Adamson, 2004; Liu, 2005a,b, 2017; Lo Bianco et al., 2009; Xiong, 2012; Xiong and Peng, 2021). Findings of some studies showed that some constraints on textbook are obvious. Adamson (2002, 2004) and Yu (2004) investigated some Chinese EFL textbooks produced in the 1970s and found that Western cultures were barely represented in textbooks. There were no texts dealing with a foreign theme or a foreign culture (Tang, 1983). There were some translation products of thoughts or slogans of the “Great Cultural Revolution,” but not even a foreign name could be found in these textbooks, let alone the Western religions.

Subsequently, this situation changed at the turn of the 21st century. Orton (2009) conducted a systematic comparative study on the textbooks used between the 1980s and those in the early years of the 21st century. Data from Orton’s study indicated a greatly expanded notion of “the world” and a considerable narrowing of the separation between Chinese and other speakers of English when compared to the situation 25 years ago. However, the utilitarian view that English was a tool for diplomatic or business purposes was still noticeably evident.

The 2010s saw the openness to a new level. Some studies investigated the cultural representations in English language textbooks. Surprisingly, these findings suggested that American and British cultures are over-represented in textbooks of mainland China, Taiwan, and Hong Kong (Yuen, 2011; Song, 2013; Lu, 2014; Liu et al., 2015; Su, 2016; Liu, 2017). Liu et al. (2021) study showed that most Chinese universities’ English language textbooks in question have overwhelmingly featured American and British cultures but marginalized the cultures of the rest of the world, and even Chinese cultures were rarely mentioned. From the review of the literature, it is evident that the language textbooks in China have seen dramatic changes during the past years. However, the investigation of ideology, more specifically, religions in language textbooks, has hardly ever been done.

While the language textbook research worldwide has built up a body of work problematizing the issues of gender and sexuality (e.g., Gupta and Lee, 1990; Stubbs, 1996; Ndura, 2004; Lee and Collins, 2010; Lee, 2014), political reinforcement (e.g., Liu, 2005a,b; Schneer, 2007; Gulliver, 2010; Xiong and Qian, 2012; Smith, 2021), and ethnic and racial stereotypes (e.g., Altbach, 1992; Koller and Mautner, 2004; Curdt-Christiansen, 2008; Hong and He, 2015; Tajeddin and Teimournezhad, 2015), the research on religious semantic domains in EFL/ESL textbooks is rarely found. The present study is designed to investigate whether the textbooks in question contain Western religion content, and if so, the study then delves into the religious semantic domains to see how the religion beliefs are presented. Because the study adopts a corpus linguistics approach, the findings will enable us to make claims about the objectivity of its findings and the replicability of its analyses.

### Methodological Issues

Tomlinson (2003) argued that language textbook evaluations were more “expert judgment” oriented, which will be inevitably influenced by subjective factors such as the evaluators’ personal bias, traditionalism, and their cultural backgrounds. Although it can be said that textbook analysis within applied linguistics is an established area of research from a variety of disciplines and perspectives (Risager, 2021), a critical overview of the methodological approaches to the analysis of cultural representations or ideological underpinnings in ELT textbooks gave a cautionary reminder about the extent to which the claims and the conclusions are accepted as completely valid and reliable (Weninger and Kiss, 2015).

The most prevalent approaches to the analysis of the cultural content in ELT textbooks are done through content analysis and Critical Discourse Analysis (Weninger and Kiss, 2015). According to Krippendorff (2013), content analysis, following a fairly structured and systematic design, requires a close reading of the data to construct a coding framework and makes inferences from texts to the contexts of their use. Manual content analysis has its strengths in qualitative analysis. It is, however, very demanding when researchers have to deal with large volumes of data (e.g., 40 volumes of textbooks in our study, as reported in this article). Therefore, this approach may “limit researchers from examining more textbooks to see a whole picture of a country’s textbooks” (Liu et al., 2021, p. 4).

The methodological issue with content analysis and CDA in some studies has also been questioned both for its reliability and data representativeness. Widdowson (2004) argued that “cherry-picking” practice was evident. That is, researchers chose data to prove a preconceived point, while inconvenient data or those that could have contradicted their “potential findings” may have been overlooked. This practice is regarded as restricting its generalizability, because some “researchers search their data for examples of what they are trying to prove, instead of letting their data ‘speak”’ (Rogers et al., 2005, pp. 379, 380). We think that corpus-assisted discourse analysis may provide an advantage in dealing with large amounts of data and in minimizing subjectivity for the following reasons.

(1) It is capable of analyzing large-scale data. Corpus linguistics has provided analysts with techniques to mine large data volumes, thus, is frequently used in the critique of newspaper articles of decades. Hunston (2002) argued that mining has become a significantly necessary undertaking for applied linguists if we are really interested in understanding language learning and teaching, and in this process, corpus linguistics has a unique role to play. Mautner (2009) also pointed out that “corpus linguistics allows critical analysts to work with much larger data volumes than they can while using purely manual techniques” (p. 123). The use of computerized analysis tools potentially strengthens the reliability of results and improves the efficiency of data analysis.
(2) The corpus-assisted text analysis employs technical corpus linguistic tools, such as Keywords and Key semantic domain in the present study, to identify linguistic features, which allows macroscopic analysis to inform the analysis at microscopic level by suggesting those linguistic features which should be investigated further. This approach can guard against “cherry-picking” practice and can provide an objective description of the data. The principles of representativeness, sampling, and balance and corpus-driven techniques, such as Keywords, help researchers to avoid over-focusing on atypical aspects of texts, “letting the data speak,” rather than writing a covert polemic (Baker and McEnery, 2015, p. 5). This corpus-assisted discourse analysis has been recognized in an increasing number of studies (e.g., Coffin and O’Halloran, 2010; Baker et al., 2013; Partington et al., 2013; Baker and McEnery, 2015; Potts, 2015; Almujaiwel, 2018). A corpus-assisted method was accordingly employed in this study to answer the research questions:
(1) Are there some religion-related semantic domains in ten sets of Chinese university ELT textbooks?
(2) If so, is there a specific authorial stance pertaining the semantic domain?

Through the review of the literature on ELT textbooks analysis and the methodological issues, the research gaps are identified in the EFL/ESL textbook analysis worldwide. The analysis aims to shed light on language textbook analysis and offers implications for the development of EFL/ESL textbooks not only in China but also worldwide. It is also an effort to draw attention to religion as a part of culture.

## Methodology

### The Research Data: University English Textbooks Corpus

A University English Textbook Corpus (UETC) was built based on 864 texts from ten current textbook series (40 volumes in total) used in China. All the textbook series included in this self-built UETC were developed and published between 2010 and 2015. They claimed to address contemporary English education requirements in China set by the Higher Education Department of Ministry of Education (2007) and the *Outline* released by the State Council. They were distributed nationally and have been adopted widely by Chinese universities for the purpose of serving Chinese educational goals with respect to the nation’s English language needs. They were approved by the Ministry of Education of the People’s Republic of China (MoE) and selected and included in the national development project for the education sector. The data related to textbooks for this study were retrieved from the UETC and analyzed through corpus-based discourse analysis. Details of the textbook series are shown in Table 1.

**TABLE 1:** Ten series of English Language textbooks.

	Textbook Name	Edition	Publishers
1	*Twenty-first Century College English* (*TCCE*)	Revision	Fudan University Press and Higher Education Press
2	*New Century College English* (*NCCE*)	2nd edition	Shanghai Foreign Language Education Press
3	*New College English* (*NCE*)	2nd edition	Shanghai Foreign Language Education Press
4	*Experiencing English* (*EE*)	3rd edition	Higher Education Press
5	*New Theme College English* (*NTCE*)	1st edition	Changchun Publishing House
6	*College English* (*CE*)	3rd edition	Peking University Press
7	*New Era Interactive English* (*NEIE*)	4th edition	Tsinghua University Press
8	*New Standard College English* (*NSCE*)	1st edition	Foreign Language Teaching and Research Press
9	*New Horizon College English* (*NHCE*)	2nd edition	Foreign Language Teaching and Research Press
10	*Top Notch College English* (*TNCE*)	1st edition	Higher Education Press

### Data Analysis

All texts in the textbook corpus were analyzed at two levels: macroscopic level in the first phase and microscopic level in the second. Richards (2001) pointed out that in corpus-based studies, qualitative analysis and quantitative assessment both were necessary and complementary, serving different purposes. The macroscopic analysis of identifying “semantic domains” is basically computer assisted quantitative analysis, which can be used to inform the microscopic level and thereby suggests those linguistic features which should be investigated further” (Rayson, 2008, p. 519) in the following phase. At macroscopic phase, Keywords and Keyword semantic domain tools of Wmatrix 4 are adopted, respectively, and simultaneously. For example, by processing the textbook corpus, the Wmatrix demonstrates the significant semantic domains which are ranked according to their log-likelihood values. Among the semantic domains, such as “Education in general,” “Language, speech and grammar,” “Science and technology,” and “Religious belief,” the researchers can focus on the analysis of the semantic domain of “Religious belief” in this case. The constituents of this domain can be demonstrated in their concordance lines, which suggests the potentially interesting language or meaning patterns for microscopic level analysis.

At the microscopic level, the analysis of the immediate context of the constituents of specific key semantic domain is a qualitative discourse analysis, and the Concordance tool of Wmatrix 4 is used to conduct context-led analysis in order to find out the semantic prosody surrounding the key notions by focusing on the use of a particular linguistic features by which some ideologies embedded, or certain authorial stance (if exists) could be unveiled.

The detailed corpus-assisted discourse analysis, along with the terminology and main concepts involved are described and illustrated as follows.

#### Macroscopic Level: Identification of Key Semantic Domains

Getting each text or corpus tagged is the first step to semantic analysis. Wmatrix, as a major tool in the present study, is a web-based corpus analysis software which was developed by Rayson (2003). It contains corpus annotation tools, Constituent Likelihood Automatic Word-tagging System (CLAWS) and UCREL Semantic Analysis System (USAS) for grammatical and semantic analysis. Texts uploaded into Wmatrix 4 are automatically annotated. The USAS semantic tagger assigns semantic tags to words in a text that has been tagged for parts of speech using CLAWS tagger. In the Tag assignment phase, Semantic Tagger (SEMTG) sort out tag assignment on a contextual basis by adopting a number of techniques.

Once the tagging is complete, researchers must choose a reference corpus as a basis for comparison. A reference corpus can help limit the subjectivity that often attends the analysis of individual texts by providing comparative data as a check measure. Wmatrix “has BNC built into its software, offering a number of subdivisions of this larger corpus” (Collins, 2015, p. 45). In the present study, we chose the “British National Corpus (BNC) Sampler Written” as the reference corpus because the texts which constitute textbooks in question are in written form. Particularly, both the textbook corpus and BNC Sampler contain a wide range of text categories (e.g., novels, poems, argumentation, expository essay, and newspaper article) and topics (e.g., humanities, campus life, arts, science, technology, society, and sports).

In corpora comparison, the keyness (or difference between corpora) in Wmatrix is determined by the default statistical measure – Log-likehood (LL). The LL is calculated by constructing a contingency table that takes four variants into account: the size of the two corpora, the frequency of the word in two corpora (see Rayson, 2008 for details of the table and formula). By comparing the tagged data of the textbook corpus and that of the reference corpus, the overused and underused semantic domains can be obtained. Semantic domain, field, or category refers to some thematic categories of keyness. They are key because of their unusual frequency in comparison with a reference corpus of an appropriate kind. For illustrative purposes, the text *Fast Food Invasion* from UETC is fed into Wmatrix 4, and the datasets are obtained from the semantic output, as shown in Table 2.

**TABLE 2:** Key semantic domains of *Fast Food Invasion*.

Rank	USAS tag	Freq	%	RC. freq	RC.%	Over/under used	LL	Semantic domain
1	F1	51	11.62	2,974	0.31	+	270.43	Food
2	I2.2	13	2.96	2,738	0.28	+	37.50	Business: selling
3	B2+	5	1.14	195	0.02	+	30.41	Healthy
4	I1.3–	2	0.46	24	0.00	+	16.72	Cheap
5	E2+	6	1.37	1,372	0.14	+	16.42	Like
6	A13	1	0.23	0	0.00	+	15.40	Degree
7	N3.2–	2	0.46	97	0.01	+	11.32	Size: small
8	Z1	1	0.23	16,434	1.70	-	8.88	Personal names
9	Z2	15	3.42	14,502	1.50	+	7.89	Geographical names

The table above shows that the leftmost column is USAS tag (or Semantic domain) ratings ranked by LL value to show key items at the top, and the two columns on the right of USAS tag column are frequency (Freq.) and relative frequency (Rel Freq.) of the USAS tags in the observed text (textbook texts). The two extra right-hand columns are the frequency and relative frequency of USAS tags in the reference corpus. Signs such as a plus (+) indicates overuse, and a minus (–) indicates underuse in the observed text relative to the reference corpus.

The LL column represents log-likelihood value on which the table is sorted. The rightmost column is the Semantic domain (field/cloud/category), which displays the most significantly overused or underused semantic categories. Key categories offer a different perspective on semantic fields as a whole, but also indicate the constituent terms within the domain.

The constituents of these categories can be demonstrated in their concordance lines. For 1 d. f., at *p* < 0.01, the cut-off 6.63 LL value would indicate that 9 USAS tags include 8 tags overused and 1 tag underused. At the *p* < 0.0001 level, the LL value is 15.13, giving 6 significant USAS tags. The cut-off point 15.13 (relates to *p* < 0.0001) is set in the present study, while items equals or above *p* < 0.01 the cut-off of 6.63 LL value was taken into account if necessary.

The most significant difference (LL value 270.43) is the semantic domain *Food* (F1). This semantic domain contains constituent words such as *fast food*, *restaurant*(*s*), *Fried*, *Yogurt*, *sandwich*, *patty*, *pie*, *pork*, *food*(*s*), *menu*, *pizza*, *burger*, *hamburger*, *diner*, *meal*, *eat*, *eating*, and *ice cream*. Clearly, most of these constituents fall into two categories: foreign food and local food, and they are collectively reflecting the topic *Fast Food Invasion*.

**FIGURE 1:**
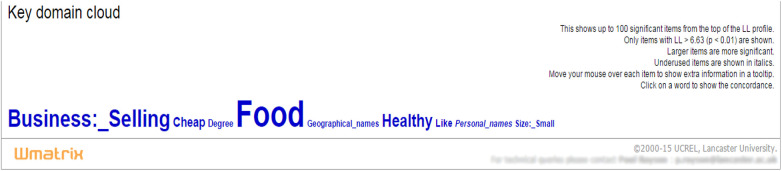
Key semantic domains for the text, *Fast Food Invasion*.

We may not be conscious of the significant semantic categories at a numerical level but when they are made known through key semantic cloud, they may validate our perception of the differences. As Collins (2015, p. 50) has argued, “[i]nsights that can be offered by such computational approaches … might not be apparent to us on first reading but …seem agreeable when supported by numerical data.” The distribution of key semantic domains is visible on the page with the constituent words under a specific semantic domain accessible by clicking it as shown in Figure 1. The larger the items present, the more significant the difference they indicate. Underused items are shown in italics. Semantic cloud gives a visual indication of the degree of the significance; the larger the size of the words, the more prominent meaning they represent.

The fact that statistical semantic key fields/domains/clouds and keywords are derived by computational measures means that they remove *a priori* biases of the analyst from the identification of themes of significance and interest (Baker, 2004; Rayson, 2008). Key semantic domains provide a starting point for pinpointing themes and issues prominent in texts and are thus worthy of further investigation at the level of discourse (Hunt and Harvey, 2015). Therefore, Wmatrix was used in this study to explore some significant semantic fields or semantic features in the textbooks in questions.

#### Microscopic Level: Concordance Analysis

More details of textual quality and in-depth meaning were examined at the microscopic level by analyzing the concordance lines of potentially interesting words. Conducting supplementary concordance and collocational analysis can enable researchers to obtain a more accurate picture of how constituents of specific semantic domain function in texts. A concordance program searches through textbook corpus and then presents a concordance display with the constituent words of specific semantic domain in the center, showing the co-text of them. Observation can be conducted both horizontally for micro context and vertically for the flow of semantic prosody.

It is generally believed that writing is an activity for specific purposes or the expressing of ideas. Successive judgment, evaluation, and authorial stance are presumed to be discerned around the words (e.g., keywords) which are closely related to the topic. If people and things were repeatedly talked about in certain ways, then it was plausible that this would affect how they were thought about (Bernstein, 1975). The concordance of keywords can provide both rich contextual information, which illuminates meaning horizontally, and characteristic usage that are vertically repeated within the text(s) studied. The cumulative products of the recurrent textual patterns indicate the semantic or attitudinal flow across a text or text groups. These specific textual functions and attitudinal flows or prosody are what the writers want most to convey. Consequently, an authorial stance is built up as the reader progresses through a text or texts.

## Findings and Discussion

### Key Semantic Domains

**FIGURE 2:**
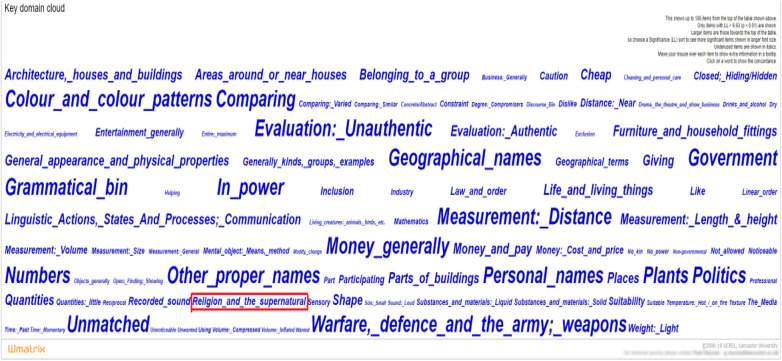
Key underused domain cloud of ten sets of textbooks.

For 1 d.f., at the *p* < 0.0001 level, the critical value is 15.13, giving 220 significant USAS tags with 125 overused and 95 underused. In this study, we focus on the underused semantic domain. Thus, the Wmatrix visualization of the resulting key domain cloud is shown in Figure 2. In the profile, larger items are more significant.

It can be seen from Figure 2 that the top significant difference in the semantic comparison is for semantic domains such as “*Warfare*, *defense and the army; weapons*,” “*Color and color patterns*,” “*Geographical names*,” and “*Money*.” Meanwhile, the figure also shows that the semantic domain of “*Religion and the supernatural*” is relatively evident though it is not among the top significant ones. The occurrence of the semantic domain “*Religion and the supernatural*” is a surprising finding.

Religion is a highly unusual topic for a Chinese foreign language textbook, given that China is a socialist country, with the Communist Party formally endorsing atheism. And atheism is also at the center of Chinese intellectual tradition, which has been established and constantly preserved for thousands of years as discussed in the introduction. This secular sociopolitical culture may thus continue to influence English education in China with regard to educational convention and socio-political tradition (Chilton et al., 2012; Sandby-Thomas, 2014). Several important aspects of atheism are as follows:

a) The Neo-Confucians did not recognize a creator or almighty God in the universe. Instead, they believed that the growth of creatures is by *Li* (force) or “natural law.”
b) They recognized the existence of *Hsin* (mind or conscience), which is somewhat comparable to the soul of Christianity, but they did not believe that this mind or conscience is bestowed by God.
c) They acknowledged that every human being has the power and the free will to reach his best state of development, to be free from sin or crime, and without God’s help to go to Heaven (Teng and Fairbank, 1954, p. 12).

However, the evidence shows that various concepts related to religion are evident in textbook corpus. The word information profile demonstrates that the total frequency of the words captured in the semantic domain *Religion and the supernatural* is up to 1,418. This category contains top frequency words such as *religion/religions/religious* (Freq. 116), *God/Gods* (Freq. 110), *Bible/Bibles/biblical* (Freq. 39), *catholic/catholicism/catholics* (Freq. 14), *Christian/Christianity/Christians* (Freq. 12), *church/churches/churchmen* (Freq. 32), *jew/jews/jewish* (Freq. 36), and *moslem/moslems/muslim/muslims* (Freq. 16), and a part of the semantic members is shown in Table 3.

**TABLE 3:** The constituents of *Religion and the Supernatural*.

Word	Semtag	Frequency	Relative frequency	
God	S9	92	0.01	**Concordance**
Religious	S9	69	0.01	**Concordance**
Spirit	S9	68	0.01	**Concordance**
Christmas	S9	51	0.01	**Concordance**
Religion	S9	40	0.01	**Concordance**
Soul	S9	35	0.01	**Concordance**
Bible	S9	31	0.00	**Concordance**
Fate	S9	30	0.00	**Concordance**
Dragon	S9	29	0.00	**Concordance**
Church	S9	27	0.00	**Concordance**
Myth	S9	27	0.00	**Concordance**
Heaven	S9	24	0.00	**Concordance**
Magic	S9	23	0.00	**Concordance**
Miracle	S9	22	0.00	**Concordance**
Spiritual	S9	22	0.00	**Concordance**
Jewish	S9	21	0.00	**Concordance**
Sacrifice	S9	18	0.00	**Concordance**
Myths	S9	18	0.00	**Concordance**
Thanksgiving	S9	17	0.00	**Concordance**
Legend	S9	15	0.00	**Concordance**
Prayer	S9	13	0.00	**Concordance**
Spirits	S9	13	0.00	**Concordance**
Catholic	S9	12	0.00	**Concordance**
Magical	S9	12	0.00	**Concordance**
Jews	S9	12	0.00	**Concordance**
Souls	S9	11	0.00	**Concordance**
Astrologers	S9	11	0.00	**Concordance**
Nun	S9	10	0.00	**Concordance**
Nuns	S9	10	0.00	**Concordance**
Ritual	S9	10	0.00	**Concordance**
Christianity	S9	10	0.00	**Concordance**
Astrologers	S9	9	0.00	**Concordance**
Blessed	S9	9	0.00	**Concordance**
Pilgrims	S9	9	0.00	**Concordance**
Sacred	S9	9	0.00	**Concordance**
Hindu	S9	9	0.00	**Concordance**
Heavens	S9	8	0.00	**Concordance**
Advent	S9	8	0.00	**Concordance**
Hell	S9	8	0.00	**Concordance**
Gods	S9	8	0.00	**Concordance**
Religions	S9	7	0.00	**Concordance**
Fable	S9	7	0.00	**Concordance**
Superstitions	S9	7	0.00	**Concordance**
Holy	S9	7	0.00	**Concordance**
Paradise	S9	7	0.00	**Concordance**
Angels	S9	7	0.00	**Concordance**
Requiem	S9	7	0.00	**Concordance**
Sacrifices	S9	7	0.00	**Concordance**
Saint	S9	6	0.00	**Concordance**
Christians	S9	6	0.00	**Concordance**
Superstition	S9	6	0.00	**Concordance**

The simple statistical analysis shows that the textbook corpus includes a variety of words relating to the concept of religion and the supernatural things, which indicates that relevant topics are discussed in texts of these textbooks. The authorial stance toward the topic, however, remains unclear. Some semantic patterns of evaluation are usually proposed by the authors in parts of a text to build up a particular evaluative position throughout the text (Martin and Rose, 2003). An in-depth analysis is needed to account for the way in which patterns of evaluative meaning accumulate through texts, and the way in which such meanings are expressed both directly and indirectly.

To reveal the authorial stance is to capture the way in which the language of a text can dynamically position the readers to follow a particular evaluative stance toward the subject of the content. Readers can be directly or indirectly channeled to view and to evaluate social and political reality in particular ways. In this regard, concordance analysis is proposed as a useful tool for critical discourse analysts to identify how the positioning of power in a text is established and built up.

The concordance profile shows that some member words relate to religions or religious concepts, but they are not the focus of the texts. For example, words “catholic/catholicism/catholics” are sporadically mentioned in different texts as “He married a Catholic girl,” and thus, these words are not the focus of the text. A close examination of the concordance profile identifies that the member word for the concept of “religion” appears frequently in three texts: *Holy Family Values*, *Literature from the Bible*, and *Can You Believe in God or Revolution*, and their semantic domains of each text are illustrated in the Tables 4–6.

**TABLE 4:** Key semantic domains for the text, *Holy Family Values*.

Item	01	%1	02	%2		LL	LogRatio	
S4	45	3.86	4002	0.41	+	120.23	3.22	Kin
S9	36	3.08	3016	0.31	+	100.01	3.31	Religion and the supernatural
A11.1+	14	1.20	2803	0.29	+	18.52	2.05	Important
S7.1-	7	0.60	697	0.07	+	17.31	3.06	No power
Z1	41	3.51	16434	1.70	+	17.24	1.05	Personal names
N5—	3	0.26	65	0.01	+	15.90	5.26	Quantities: little
A13	1	0.09	0	0.00	+	13.44	10.70	Degree
S2.2	11	0.94	2534	0.26	+	12.27	1.85	People: male
G2.2-	5	0.43	516	0.05	+	12.05	3.01	Unethical
Z8	121	10.37	72023	7.44	+	11.98	0.48	Pronouns
Q2.1	20	1.71	7024	0.73	+	11.30	1.24	Speech: communicative
W1	6	0.51	912	0.09	+	10.54	2.45	The universe
L1-	7	0.60	1585	0.16	+	7.99	1.87	Dead
L1+	2	0.17	93	0.01	+	7.71	4.16	Alive
A6.2+	8	0.69	2275	0.23	+	6.60	1.54	Comparing: usual
G2.2+	3	0.26	405	0.04	+	5.86	2.62	Ethical

**TABLE 5:** Key semantic domains for the text, *Literature from the Bible*.

Item	01	%1	02	%2		LL	LogRatio	
S9	40	4.77	3,016	0.31	+	143.03	3.94	Religion and the supernatural
Q3	24	2.86	1,653	0.17	+	89.86	4.07	Language, speech, and grammar
Q4.1	17	2.03	1,741	0.18	+	51.23	3.49	The Media: books
Q4	7	0.83	740	0.08	+	20.69	3.45	The Media
X9.2++	1	0.12	0	0.00	+	14.10	11.17	Success
A13	1	0.12	0	0.00	+	14.10	11.17	Degree
A13.2	7	0.83	1,439	0.15	+	12.62	2.49	Degree: maximizers
Q1.2	10	1.19	3,691	0.38	+	9.18	1.64	Paper documents and writing
A4.2–	1	0.12	9	0.00	+	7.62	7.00	General
A5.1+	8	0.95	2,905	0.30	+	7.52	1.67	Evaluation: good

**TABLE 6:** Key semantic domains for the text, *Can You Believe in God or Revolution*.

ITEM	01	%1	02	%2		LL	LogRatio	
S9	38	3.17	3016	0.31	+	107.40	3.35	Religion and the supernatural
L1+	13	1.08	93	0.01	+	95.41	6.82	Alive
Y1	18	1.50	778	0.08	+	70.95	4.22	Science and technology in general
A13	3	0.25	0	0.00	+	40.17	12.24	Degree
X2.1	22	1.83	4139	0.43	+	30.28	2.10	Thought, belief
A2.1+	18	1.50	3939	0.41	+	20.72	1.88	Change
W1	8	0.67	912	0.09	+	17.53	2.82	The universe
N5—	3	0.25	65	0.01	+	15.74	5.22	Quantities: little
W5	4	0.33	225	0.02	+	13.81	3.84	Green issues
A6.2+	11	0.92	2275	0.23	+	13.57	1.97	Comparing: usual
S2	12	1.00	2896	0.30	+	12.14	1.74	People
A5.2+	6	0.50	779	0.08	+	11.83	2.64	Evaluation: true
X2.5+	5	0.42	551	0.06	+	11.25	2.87	Understanding
G2.2	3	0.25	147	0.02	+	11.12	4.04	General ethics
X9.1+	6	0.50	1060	0.11	+	8.84	2.19	Able/intelligent
X7+	15	1.25	5233	0.54	+	8.13	1.21	Wanted
X4.1	8	0.67	1947	0.20	+	8.00	1.73	Mental object: conceptual object
A3+	46	3.84	24177	2.50	+	7.38	0.62	Existing
Q3	7	0.58	1653	0.17	+	7.29	1.77	Language, speech and grammar

It can be seen from Tables 4–6 that the most significant difference in the semantic comparison is for the tag S9, representing the semantic field *Religion and the Supernatural*, which verifies the global quantitative assessment. This suggests the linguistic features which should be further investigated to explore the authorial stance. Our study moved to microscopic analysis for context-led analysis in order to find out the authorial stance. The concordance profile provides an analyst a privileged viewpoint on patterns of co-selection by examining horizontal co-text with the node word aligned in the center, and a viewpoint on patterns of repetition by looking at vertical paradigmatic features of the node word. A recurrent string of part of the text is directly observable.

### Semantic Prosody and Authorial Stance

Upon examining the concordance for the tag of *Religion and the supernatural*, a positive semantic prosody is captured as shown in Table 7.

**TABLE 7:** Concordance of key domain *Religion and the Supernatural* of the text, *Holy Family Values*.

	Holy	Family Values Sometime around the beginning of the Common Era, a nice Jewish girl comes to
Holy Family Values Sometime around the beginning of the Common Era, a nice	Jewish	girl comes to her fianc with a problem. She is pregnant; he is not the father.
as only beginning. The righteous man, Joseph, goes to sleep and receives a visit from an	Angel	. “Joseph, son of David,” the angel says, do not be afraid to take
to sleep and receives a visit from an angel. “Joseph, son of David,” the	Angel	says, do not be afraid to take Mary home as your wife, because what is conceived in her
not be afraid to take Mary home as your wife, because what is conceived in her is from the	Holy Spirit	. She will give birth to a son and you arc to give him the name Jesus for
to give him the name Jesus for he will save his people from their sins. Like all good	Jews	who had received visits from God or angels before him Abraham, Moses Joseph docs as he is
he will save his people from their sins. Like all good Jews who had received visits from	God	or angels before him Abraham, Moses Joseph docs as he is told. The baby is born in
save his people from their sins. Like all good Jews who had received visits from God or	Angels	before him Abraham, Moses Joseph docs as he is told. The baby is born in Bethlehem;
baby is born in Bethlehem; his human parents name him Jesus. As the world’s 2 billion	Christians	prepare to commemorate the birth of the figure they believe to be the Son of God, it is
Christians prepare to commemorate the birth of the figure they believe to be the Son of	God	, it is important to note that Christianitys origins lie more in the image of the empty tomb
the birth of the figure they believe to be the Son of God, it is important to note that	Christianitys	origins lie more in the image of the empty tomb on the Sunday after the crucifixion than the
Sunday after the crucifixion than they do at the creche. It was their fervent belief in the	Resurrection	of Jesus of Nazareth that convinced his followers he was, as Peter put it, the Christ,
that convinced his followers he was, as Peter put it, the Christ, the son of the living	God	“who had told them of a new way of salvation: that he would die and rise again,
, the Christ, the son of the living God” who had told them of a new way of	Salvation	: that he would die and rise again, thus effecting the forgiveness of sins and offering
and offering a portal to eternal life. But whatever ones personal beliefs, no student of	Religion	or culture should overlook the significance of the world of the Nativity, for the milieu
no student of religion or culture should overlook the significance of the world of the	Nativity	, for the milieu into which Jesus was born and in which he was raised has fundamentally
raised has fundamentally shaped the manners and morals of the ensuing two millennia. The	Jewish	family values that were prevalent in first-century Judea the values of Mary and Joseph and
Judea the values of Mary and Joseph and of the young Jesus became the values of	Christianity	, and of the regions of the world in which Christianity has long been a critical force.
young Jesus became the values of Christianity, and of the regions of the world in which	Christianity	has long been a critical force. Jesus is the kind of Jewish son a mother would be proud
of the world in which Christianity has long been a critical force. Jesus is the kind of	Jewish	son a mother would be proud of: he takes the family values of his childhood and, in his
world is coming and what matters now is the community of believers, the followers of the	Messiah	on earth and in heaven. What matters is the family, as he put it, of man
matters now is the community of believers, the followers of the Messiah on earth and in	Heaven	. What matters is the family, as he put it, of man. Throughout the Gospels
heaven. What matters is the family, as he put it, of man. Throughout the	Gospels	, Jesus makes this point again and again. Let the dead bury the dead,” he says
goodbyes. The only thing a believer must do is follow me” and proclaim the Kingdom of	God	. The meaning of this message was powerful to his followers, who tended to be laborers and
your mother, and thats extraordinary’, says Amy-Jill Levine, author of The Misunderstood	Jew	and a professor at Vanderbilt. That is why, when the Pharisees come to him and ask him
author of The Misunderstood Jew and a professor at Vanderbilt. That is why, when the	Pharisees	come to him and ask him how to punish an adulterer, he takes her side. If any
there are listeners in his hearing who shall not taste death” before the coming of the	Messiah	practical concerns about the care and feeding of kin were less than critical. Jesus
with respect of eunuchs who have made themselves eunuchs for the sake of the kingdom of	Heaven	. Let anyone accept this who can. As the years passed and the early church took shape
kingdom of heaven. Let anyone accept this who can. As the years passed and the early	Church	took shape, the original followers began, naturally, to die off, and the followers of
to make: would they shape the message for the masses or would they opt, like Paul and the	Jewish	Essenes, for a more ascetic approach? The decision was urgent, for history was unfolding
be doomed. It was, then, time to return to the values of the world of the	Nativity	, to ways of preserving families even in times of crisis. In her 1988 book Adam, Eve
asserts the commandment Honor your mother and father. Near the end of the first century.	Pope	Clement argues, Jesus unmarried state was in no way meant to be an example for everyone:
marry was that, in the first place, he was already engaged, so to speak, to the	Church	; and, in the second place, he was no ordinary man. No matter what one thinks
no ordinary man. No matter what one thinks of Jesus of Nazareth that he was the Son of	God	, an interesting prophetic figure or a religious provocateur with particularly prolific
of Jesus of Nazareth that he was the Son of God, an interesting prophetic figure or a	Religious	provocateur with particularly prolific followers surely we can agree that he was no ordinary
end of his life, then, Jesus took care of his mother, the penultimate act of a nice	Jewish	boy and a blessing of the kinds of values that should endure, as his followers say even now

The profile of concordance lines is extracted from the textbook corpus. As shown by the concordance lines, the lexical items that co-occur with the constituents of *Religion and the supernatural* are *all good Jew*, *nice Jewish boy*, *significance of*…*Nativity*, *Christianity has long been a critical force*, *Jewish son a mother would be proud of*, *preserving families*, *honor your mother and father*, *son of God an interesting prophetic figure*, *blessing values that should endure*, etc. Looking at their immediate context, we can see that most of these co-occurrences create a positive semantic prosody, and there is no consistent negative authorial stance toward Western religions. The similar textual and linguistic characteristics happen to the text *Literature from the Bible* as follows.

The concordance tables, Tables 7, 8, present an overview of the immediate context surrounding the key node words and most of the co-occurrences are represented by a positive or neutral evaluative language; no negative linguistic patterns are evident. For example, *the Bible*…*serve as a valid and exciting introduction to literature*, *Bible has been a keystone in the arch of great books*, *Bible is both the all-time best-seller*, *and*, *by far*, *the most translated book*, *significant Bible*, and *magnificent translation*, among others. It can be argued that the lexical items that co-occur with member words of religion and supernatural in two texts above created a somewhat vertical groove of positive semantic prosody toward religion and the related concepts.

**TABLE 8:** Concordance of key domain *Religion and the Supernatural* of the text *Literature from the Bible*.

	40 occurrences.	
Literature from the	Bible	The word Bible comes from the Greek biblia, meaning little books, and the Bible is, literally,
Literature from the Bible The word	Bible	comes from the Greek biblia, meaning little books, and the Bible is, literally, a collection of
the Bible The word Bible comes from the Greek biblia, meaning little books, and the	Bible	is, literally, a collection of little books. The earliest parts of some of them go back
beginning with “Beowulf” and ending with an author of the twentieth century. The	Bible	is also a selective sampling of Hebrew literature. As such it contains a wide range of writing:
Hebrew literature. As such it contains a wide range of writing: folk tales,	Legends	, sagas, fables, riddles, songs, history, philosophy, short stories, biography and this list
As such it contains a wide range of writing: folk tales, legends, sagas,	Fables	, riddles, songs, history, philosophy, short stories, biography and this list is not complete.
short stories, biography and this list is not complete. Therefore, the	Bible	, quite apart from its theological and cultural importance, can serve as a valid and exciting introduction to the
and this list is not complete. Therefore, the Bible, quite apart from its	Theological	and cultural importance, can serve as a valid and exciting introduction to the study of literature. Furthermore
with a list of suggested readings that can, collectively, point up how the	Bible	has been a keystone in the arch of great books. Understandably, the Bible is both the all-time
how the Bible has been a keystone in the arch of great books. Understandably, the	Bible	is both the all-time best-seller and by far the most translated book in the world. For English-speaking people
completed in 1611. It is this edition which Milton and Bunyan knew, which the	Pilgrims	brought with them to Massachusetts, which American pioneers earned westward. Consequently it is the edition that
English and American thought and expression. Under Henry’ VIII, when the English	Church	broke away from Rome, one of the immediate and urgent needs was for a Bible printed in the native
English church broke away from Rome, one of the immediate and urgent needs was for a	Bible	printed in the native language. In the 1520s and 1530s William Tyndale, working from the Greek text
from the Greek text of Erasmus, Luther’s German edition, and the traditional Latin	Bible	, published on the Continent his translations of most of the Bible. Much of the diction and rhythm
traditional Latin Bible, published on the Continent his translations of most of the	Bible	. Much of the diction and rhythm, as well as many of the familiar phrases of the King
Much of the diction and rhythm, as well as many of the familiar phrases of the	King James Version	, had been taken from Tyndale, and it is to him that the English Bible is deeply
King James Version, had been taken from Tyndale, and it is to him that the English	Bible	is deeply indebted for the forceful and often colloquial nature of its sentence structure and vocabulary. In 1535
colloquial nature of its sentence structure and vocabulary. In 1535 the first complete	Bible	printed in English appeared. Its “author,” Miles Coverdale, relied heavily on Tyndale but removed
of his own that have become part of our linguistic heritage. Two other significant	Bibles	followed. The Geneva Bible, published in 1560, was for half a century the translation used in
part of our linguistic heritage. Two other significant Bibles followed. The Geneva	Bible	, published in 1560, was for half a century the translation used in most English homes, and,
used in most English homes, and, next to Tyndale’s, it was the book from which	King James Version	borrowed most heavily. In all probability this was the version Shakespeare read, and it was
this was the version Shakespeare read, and it was the edition favored by the early	Puritans	. Then in 1568 came the Bishops’ Bible, a large and costly volume that, as the
read, and it was the edition favored by the early Puritans. Then in 1568 came the	Bishops	‘Bible, a large and costly volume that, as the official Bible of the Church of England,
it was the edition favored by the early Puritans. Then in 1568 came the Bishops’	Bible	, a large and costly volume that, as the official Bible of the Church of England, likewise contributed
in 1568 came the Bishops’ Bible, a large and costly volume that, as the official	Bible	of the Church of England, likewise contributed to its replacement. Paradoxically, this magnificent translation
and economic disputes in 17th-century England were so interlocked with problems of	Theology	that religion was not something remote; and the vocabulary of religion of, explicitly, the Protestant Bible was
disputes in 17th-century England were so interlocked with problems of theology that	Religion	was not something remote; and the vocabulary of religion of, explicitly, the Protestant Bible was used in
problems of theology that religion was not something remote; and the vocabulary of	Religion	of, explicitly, the Protestant Bible was used in forum, market place, and parlor, not just
was not something remote; and the vocabulary of religion of, explicitly, the	Protestant	Bible was used in forum, market place, and parlor, not just in the pulpit. Second
something remote; and the vocabulary of religion of, explicitly, the Protestant	Bible	was used in forum, market place, and parlor, not just in the pulpit. Second,
committee was not starting from scratch. To create this new Authorized Version, 50	Churchmen	, working in 6 groups, spent 6 years revising earlier English Bibles. Two members from each group
Version, 50 churchmen, working in 6 groups, spent 6 years revising earlier English	Bibles	. Two members from each group then acted as a board of review, and from them the copy
of review, and from them the copy went through a final check at the hands of two	Bishops	. Not only was the committee’s work scholarly and collective, but the final result represented a compromise
scholarly and collective, but the final result represented a compromise between various	Doctrinal	views. Yet the King James Version was almost immediately recognized as a classic, and it may well
the final result represented a compromise between various doctrinal views. Yet the	King James Version	was almost immediately recognized as a classic, and it may well represent the rare instance of a
it must be reinterpreted by each generation; otherwise it dies. The fact that the	Bible	is very much alive raises the question, however, of how and why the ancient writings of a small
should be relevant to our own massive, fast-changing world. One answer is that the	Bible	is the word of God. Equally coherent is the answer that the Bible is a record of Hebrew
our own massive, fast-changing world. One answer is that the Bible is the word of	God	. Equally coherent is the answer that the Bible is a record of Hebrew history and their hopes and
answer is that the Bible is the word of God. Equally coherent is the answer that the	Bible	is a record of Hebrew history and their hopes and desires and the early Christian church was formulated by men
is a record of Hebrew history and their hopes and desires and the early Christian	Church	was formulated by men who examined and expressed both what is meant and means to be human, and was

The key semantic domains of the text *Can You Believe in God or Revolution* in Table 7 presents that the top two semantic domains of substantial meaning are *Religion and the supernatural* and the other is *Science and technology in general*, which verifies the topic reflected in the text title. Other significant semantic fields “*Change*,” “*Thought*, *belief*,” “*Comparing: Usual*,” “*Evaluation: True*,” “*Mental object: Conceptual object*” may, to different degrees, reveal the content and authorial attitude. However, a further examination on the expanded local context is expected to unveil the way in which the author builds his or her stance in the text flow.

The text *Can You Believe in God and Evolution* projects four outstanding intellectuals’ distinctive viewpoints and values on “God” and “evolution,” as shown in the following excerpts:

a) Francis Collins, Director, National Human Genome Research Institute
*I see no conflict in what the Bible tells me about God and what science tells me about nature*…*I lead the Human Genome Project*, *which has now revealed all of the 3 billion letters of our own DNA instruction book*. *I am also a Christian*. *For me*, *scientific discovery is also an occasion of worship*… *Science’s tools will never prove or disprove God’s existence*. *For me*, *the fundamental answers about the meaning of life come not from science but from a consideration of the origins of our uniquely human sense of right and wrong*, *and from the historical record of Christ’s life on Earth*.
b) Steven Pinker, a Psychology Professor, Harvard University
*Many people who accept evolution still feel that a belief in God is necessary to give life meaning and to justify morality*. *But that is exactly backward*. *In practice*, *religion has given us stonings*, *inquisitions*, *and 9/11*. *Morality comes from a commitment to treat others as we wish to be treated*, *which follows from the realization that none of us is the sole occupant of the universe*. *Like physical evolution*, *it does not require a white-coated technician in the sky*.
c) Biochemistry professor, Lehigh University, Senior fellow, Discovery Institute
*Sure*, *it’s possible to believe in both God and evolution*. *I’m a Roman Catholic*, *and Catholics have always understood that God could make life any way he wanted to*. *If he wanted to make it by the playing out of natural law*, *then who were we to object? We were taught in parochial school that Darwin’s theory was the best guess at how God could have made life*… *I’m still not against Darwinian evolution on theological grounds*. *I’m against it on scientific grounds*. *I think God could have made life using apparently random mutation and natural selection*.
d) Albert Mohler, President, Southern Baptist Theological Seminary
*There are people who will say they hold both positions*. *But you cannot coherently affirm the Christian truth claim and the dominant model of evolutionary theory at the same time*… *I think it’s interesting that many of evolution’s most ardent academic defenders have moved away from the old claim that evolution is God’s means to bring life into being in its various forms*. *More of them are saying that a truly informed belief in evolution entails a stance that the material world is all there is and that the natural must be explained in purely natural terms*. *They’re saying that anyone who truly feels this way must exclude God from the story*.

The textbook authors/compliers/writers have obviously adopted an eclectic approach in terms of material selection. The “voices” of people in this text are from different professions with different roles, but their distinctive views are equally presented. The genome researcher is a Christian, believing that a faith both in religion and in science is possible; a Harvard psychology professor questions religion from the perspective of morality; a Roman Catholic finds theological grounds for Darwinian evolution and God; a Baptist theological seminary objects to the position of the coexistence of God and evolution.

Obviously, there is no certain consistent stance captured in the text, but varied views on the concepts of religion and evolution are evident. In addition, in this text, no evaluative comments favoring any argument were found. It appears that there is no purposeful manipulation of materials when selecting and adapting pre-existing ideologies or any evidence of prejudice against religious beliefs in this textbook text.

The empirical findings in this study provide a new understanding of Chinese foreign language textbooks in the new era, though, these findings are inconsistent with what Zhang and Pérez-Milans (2019) reported in their language policy study. In their view, education for secondary schools in the Tibetan region does not allow for religious contents in “constructing Tibetan language education as a pedagogical space” (Zhang and Pérez-Milans, 2019, p. 39). As Luke (1988) argued, the curriculum of education is not an ideological reflection. Rather, it is a complex historical dynamic in the transmission and maintenance of a selective tradition. It would be of little help to try to interpret certain social values or cultural representations only by concentrating on the discursive linguistic items or teaching practice. The positioning of religion in Chinese education may be better understood both from the perspective of socio-cultural considerations and of political progress.

## Conclusion

The purpose of the current study was to investigate the religious cultural representation and the authorial stance toward the concept of religion in the university English textbooks. The discourse analysis of the 10 sets of Chinese universities’ English language textbooks, using corpus-linguistics tools has shown that a variety of words relating to the concept of religion and the supernatural are demonstrated in the textbook corpus. Western religious beliefs-related frequency words include *God/Gods*, *Bible/Bibles/biblical*, *catholic/catholicism/catholics*, *Christian/Christianity/Christians*, *church/churches/churchmen*, *Jew/Jews/Jewish*, and *Moslem/moslems/muslim/muslims*. Western religion is a highly unusual topic and has never appeared in Chinese English language textbooks before because of the political and the socio-cultural constraints in Chinese history. While the semantic domain of “*Religion and the supernatural*” is not significantly represented, its appearance in mainstream university English textbooks is an indication that the Chinese foreign language textbook development has seen dramatic changes since the 2020s.

It is noteworthy that when presenting the views and the attitudes toward Western religious beliefs, textbook writers or compliers have adopted an eclectic approach to materials selection. Additionally, the texts in these textbooks represent distinctive views. Regarding the authorial stance, some positive and favorable semantic prosodies surrounding the member words of the concept of religion are captured, and there is no consistent, evident negative authorial stance toward religion though atheism is in the center of Chinese intellectual tradition for thousands of years.

This research also provides a framework for the exploration of cultural representation and ideology with a corpus-assisted discourse analysis approach. In this methodology, several practical applications of some corpus linguistics tools are employed to holistically identify the research topic from a large volume of data at the global level of all the texts, and to trace possible regional semantic grooves constituted by each local semantic prosody. The strengths of this methodology lie in its objectivity and replicability, which is particularly valuable in discourse analysis as applied to textbook analysis. The corpus-assisted method, however, has its limitations. Some data analyzed in this way can be interpreted but providing data does not automatically encode any social meanings for them. Still, anything more than the data must be theorized and explained, which can be a joint endeavor by the researcher and the users of these textbooks. Despite the advantage of being able to process large volumes of data, corpus linguistic tools are not human agents. Therefore, in future studies, researchers interested in using this method need to be mindful of these limitations.

## Author Biodata

YL has earned her Ph.D. from The University of Auckland, New Zealand and is currently an Associate Professor in the School of Foreign Studies at Yanshan University, China. Her research agenda involves corpus linguistics assisted discourse analysis and intercultural teaching and learning in higher education. She has published her research in *Language*, *Culture*, *and Curriculum* and some prestigious Chinese journals of applied linguistics, including *Journal of the Foreign Language World*, *Foreign Language Education in China*, and *Contemporary Foreign Language Studies*.

LZ, Ph.D., is Professor of Applied Linguistics and Teaching English to Speakers of other Languages (TESOL) and Associate Dean, Faculty of Education and Social Work, University of Auckland, New Zealand. A past Post-Doctoral Fellow at the University of Oxford, he has published extensively on the psychology of language learning and writing in *Applied Linguistics; Modern Language Journal; Applied Linguistics Review; British Journal of Educational Psychology*; *Discourse Processes; Reading and Writing; Reading and Writing Quarterly; Journal of Psycholinguistic Research; Instructional Science*; *System*; *TESOL Quarterly*; *Language Awareness; Language*, *Culture and Curriculum; Language and Education; Teachers and Teaching*, and *Journal of Second Language Writing*. His current interests lie in reading and writing development, especially the acquisition of L2 written language and EAP. He was the sole recipient of the “TESOL Award for Distinguished Research” in 2011 for his article in *TESOL Quarterly*. He is a co-editor for *System*, serving on the editorial boards of *Journal of Second Language Writing*, *Applied Linguistics Review*, *Australian Review of Applied Linguistics*, *Metacognition and Learning*, *Chinese Journal of Applied Linguistics*, and *RELC Journal*. Additionally, he reviews manuscripts for *Applied Linguistics*, *Language Learning*, *MLJ*, *Reading and Writing*, *Reading and Writing Quarterly*, and *Review of Educational Research*, *Teachers and Teaching*, *TESOL Quarterly*, among other journals.

LY, Ph.D., is a Lecturer at the School of Foreign Languages, Jiangsu University of Science and Technology, China. Having a Ph.D. from The University of Auckland, New Zealand, Francoise’s research interests include second language writing, self-regulated learning and socio-cultural contexts of English language learning and teaching.

## Data Availability Statement

The original contributions presented in the study are included in the article/supplementary material, further inquiries can be directed to the corresponding author.

## Ethics Statement

The studies involving human participants were reviewed and approved by the University of Auckland Ethics Committee on Human Participants. The patients/participants provided their written informed consent to participate in this study. Written informed consent was obtained from the individual(s) for the publication of any potentially identifiable images or data included in this article.

## Author Contributions

YL conceived of the initial idea, designed the study, collected and analyzed the data, and drafted the manuscript. LZ and LY revised the manuscript. LZ fine-tuned the initial idea and proofread and finalized the manuscript for submission as the corresponding author. All authors contributed to the article and approved the submitted version.

## Conflict of Interest

The authors declare that the research was conducted in the absence of any commercial or financial relationships that could be construed as a potential conflict of interest.

## Publisher’s Note

All claims expressed in this article are solely those of the authors and do not necessarily represent those of their affiliated organizations, or those of the publisher, the editors and the reviewers. Any product that may be evaluated in this article, or claim that may be made by its manufacturer, is not guaranteed or endorsed by the publisher.
